# An expandable voice user interface as lab assistant based on an improved version of Google’s speech recognition

**DOI:** 10.1038/s41598-023-46185-x

**Published:** 2023-11-09

**Authors:** Maria Fernanda Avila Vazquez, Nicole Rupp, Larissa Ballardt, Jeannine Opara, Thole Zuchner

**Affiliations:** Faculty for Life Sciences, Professorship for Bioanalytics and Laboratory Automation, Albstadt-Sigmaringen University, Anton-Günther-Str. 51, 72488 Sigmaringen, Germany

**Keywords:** Machine learning, Programming language, Software, Computational platforms and environments

## Abstract

Voice assistants are potentially helpful when working in a scientific laboratory. A big challenge is the extremely specific use of language in every laboratory. As with any voice assistant, another concern is data security. Here, we present Rainbow—an open source voice user interface (VUI) for scientific laboratories, that is adaptable to any Windows PC with Internet access. We used Google Translate Site (GTS) as a voice input and output system to ensure communication to the user. The scripting language AutoIt controls GTS, executes all actions and builds the VUI. Rainbow performs tasks from three different areas—general Microsoft Windows tasks, lab-specific tasks, and device-specific tasks. We achieved significantly higher speech recognition accuracy with our VUI than with GTS alone (91.3% versus 85.1%). Because of Rainbow's architecture, it is possible to improve the voice assistant in terms of functionality and accuracy, allowing each laboratory to optimize its own Rainbow system in a user-friendly way. In a test setup, this led to a speech recognition accuracy of 98.6%. Taken together, Rainbow provides an opportunity for every scientist to implement highly specific scientific terms and tasks to this open source voice assistant system in a very user-friendly way.

## Introduction

The simplest and most natural way of communication for humans is the use of language^1,2^. We can only guess the need of a crying newborn but we start to communicate and understand the child when it starts speaking in toddlerhood. Therefore, it is not surprising that developers wanted to enable humans the interaction with computer-controlled devices via voice commands. Today, communication with devices via so-called voice user interfaces (VUI) has become a matter of our everyday life^2–5^.

Since the 2010s, Apple's Siri, Amazon's Alexa, Samsung's Bixby and others have allowed us, for instance to make calendar entries while driving, turn on the lights while holding purchases in our hands, or ask about the weather while choosing the next day's outfit standing in front of the closet^3^. Thus, voice assistants have clearly improved our everyday lives, as they can perform small tasks while we are busy doing other things. Therefore, they are also highly interesting for many industrial areas-not at least in scientific laboratories^4^.

In addition to the automation of laboratory processes, improved communication and intuitive control of hardware and software components is a key component of a modern laboratory^2,7^. Therefore, the use of a VUI in the laboratory is an attractive thought^2,3,8^. Working in a laboratory usually involves following protocols and incubation times, interacting with equipment and its software, and documenting experiments—often at the same time.

Voice assistants perform these tasks by voice command, allowing employees to continue working without interruption. For example, when performing an experiment: To set a timer or take notes, the gloves should first be removed to avoid contamination of objects. When the activity is done, new gloves are put on and the main experiment can be continued. It is even more time-consuming when working under the laminar flow bench, as removing and inserting the gloved-hands into a laminar flow bench is even more time-consuming here. Although the conventional voice assistants mentioned above, such as Amazon's Alexa or Apple's Siri, perform well for daily task, they have limited utility in specialized areas like scientific laboratories. On the one hand, they show limitations in speech recognition of specialized vocabulary of different scientific fields^5^. For example we noticed, that Samsung’s Bixby has struggle with speech recognition of units like “micromole per liter”. On the other hand, they are not programmed to perform specific laboratory commands—for instance handling special equipment or calculating parameters underlying scientific calculations. Therefore, commercial voice assistants cannot simply be used in the scientific laboratory, but must at least be adapted. Manufacturers such as LabTwin, LabVoice or Elementa Labs have developed voice assistants specifically for the laboratory^9–11^. Thermo Fisher also allows the use of speech when operating a real-time PCR system^12^. Others like Austerost et al., Rhodes et al. or Hill developed so-called “skills” for the existing Amazon Alexa device specifically for the laboratory which, however, raises concerns about data privacy^2,5,13–15^.

Another challenge is the diversity of people working in the laboratory. In contrast to the population using everyday voice assistants, the target population for VUIs in the laboratory is much smaller, which makes the diversity of dialects and accents more noticeable. Hence, in laboratory environments worldwide, a comparatively small number of people with extremely varying dialects is using highly specific vocabulary which is different in almost every lab. Developing a single system that reliably understands all laboratory-specific words in all dialects therefore is an enormous challenge^14^.

The advantages of voice control—particularly in the laboratory—are indisputable, but aspects such as costs, data privacy, training requirements, and users' accents and dialects must be taken into account^8,16,17^.

As a proof-of-principle, we wanted to investigate whether free software components could be used to develop a voice control assistant for scientific laboratories. The most important ability of a voice assistant is to understand the spoken commands, to analyze them, and to execute the corresponding action^18^. We used Google's Google Translate Site (GTS) and the scripting language AutoIt to develop Rainbow—a voice assistant that performs Windows-based, scientific, and device-specific tasks via voice command. Using the AutoIt scripting language, we were able to improve the accuracy of GTS. In addition, speech recognition can be continuously improved via a Graphical User Interface (GUI) to enable Rainbow to learn English terms in a wide variety of accents and dialects. Furthermore, the flexibility of our voice assistant allows the expansion of task capabilities as needed, without increasing the risk in terms of data security.

## Materials and methods

We developed the VUI rainbow using AutoIt version 3.3.14.5 (AutoIt Consulting Ltd., Worcestershire, UK) with the corresponding AutoIt Script Editor (SciTE4AutoIt3, Neil Hodgson) version 4.4.6 and AutoIt v3 Window Information to view information that are necessary for scripting (e.g., mouse position for a mouse click or window title to communicate with a running application). For scripting, we utilized supportive materials like the AutoIt Help (v3.3.14.5) and the AutoIt Forum (www.autoitscript.com/forum/). AutoIt is compatible for Microsoft Windows XP up to Windows 11, we used a desktop computer (Intel Core i5-3470, 16 GB RAM) with operating system Window 10 Bit (Microsoft, Redmond, Washington). The main tasks of AutoIt as VUI is the menu navigation, processing of voice input and execution of the corresponding activities. For voice input and output we used Google Translator’s Site (GTS) (Google, LLC., Mountain View, California) set in English language via Google Chrome Browser (Google, LLC., Mountain View, California). Additionally, we used a Bluetooth paired Logitech Mono H820e Wireless headset (Logitech, Lausanne, Switzerland) for command input and receiving feedback from the VUI.

Rainbow allows running all installed programs on the desktop computer by voice command. However, we used Microsoft Word, Microsoft Excel, Microsoft Editor and Microsoft Edge (Microsoft, Redmond, Washington) for special activities. As analysis software, SoftMax Pro Software version 7.0.3 (Molecular Devices, San Jose, California) was integrated into the VUI to control the microplate reader SpectraMax iD5 (Molecular Devices, San Jose, California).

We invited 38 user volunteers not familiar with the VUI—but from the scientific area—for testing the VUI to obtain the system’s accuracy and reliability. The results should indicate Rainbow's suitability for everyday use with our volunteers representing a sample of potential users. All procedures including human participants were performed in accordance with relevant guidelines. According to the German Research Foundation (DFG), the governmental science funding organization in Germany, a waiver of ethics approval is not needed for this study^19^. Informed consent was obtained from all volunteers who participated in this study.

Most of the volunteers were native German speakers with Swabian dialect (n = 18). Other characteristics of the volunteers were the standard German (n = 13), Turkish (n = 2), Russian (n = 2), Hungarian (n = 1), and Spanish (n = 2) pronunciation. Fourteen of the 38 volunteers identify as male, while 24 are female in the age range between 26 and 57, with an average of 40 years (standard deviation = 11.5). The testing took place in a bioanalytical laboratory with active ventilation and other background noises, which represents usual laboratory conditions. Due to the pandemic condition, all volunteers wore a FFP2-mask during voice input. To determine whether we can expect improved GTS accuracy with rainbow than with GTS alone, we asked the volunteers to perform the test with Rainbow on the one hand and with GTS alone on the other. To cover all possible Rainbow commands, we prepared two sets of 35 or 36 commands and asked the volunteers to perform one of them. We formulated the command sets to represent a possible and meaningful conversation with Rainbow. During the accuracy tests, each volunteer wore the headset and received one of the two sets of commands. This set of commands was first pronounced by applying Rainbow and then repeated for GTS. Supplementary Table S1 contains both sets of commands with the resulting rainbow conversations.

During the accuracy tests, we documented correctly recognized and incorrectly recognized commands. Therefore, we prepared a Microsoft Excel 2016 (Microsoft Corporation) sheet with all commands per user and recorded one of two possible outcomes. For correctly recognized commands in GTS, we enter a “1”, for misrecognized commands we enter a “2”. The same applies to Rainbow; we record a “1” for correct executed functions and a “2” for errors. For statistical comparison of the results, we performed a right sided McNemar test (n = 1,349; α = 0.05) to test whether the number of improvements from GTS to Rainbow are greater than the deteriorations from GTS to Rainbow. We used RStudio statistical software (RStudio Team (2020) to execute the statistical test. RStudio: Integrated Development for R. RStudio, PBC, Boston, MA, http://www.rstudio.com/) version 2022.02.3 + 492 for macOS.

Each user can improve Rainbow's accuracy. For this purpose, we developed a graphical user interface (GUI) that allows the user to integrate incorrect commands and associate them with the correct command. The GUI was also scripted using AutoIt and acts as an add-on to Rainbow. To test the relevance of the GUI, we performed an experiment with an employee as an example. First, the employee determined the personal Rainbow accuracy by testing both sets of commands (n = 71). Then, the employee repeatedly spoke both sets of commands three times (n = 213) using GTS and noted any misrecognized commands. After that, the employee recorded relevant misrecognized commands via the GUI in Rainbow and tested Rainbow by repeating both sets of commands three times (n = 213). The success rate is expressed by the percentage of successful commands out of all commands.

## Results

Using the scripting language AutoIt and GTS we developed the VUI “Rainbow”—a voice assistant for laboratory applications. Rainbow can perform computer-based tasks from three different areas by voice, including Microsoft Windows Automation (A), Laboratory skills (B) and Analytical instrument skills (C). These areas will be explained in more detail later in this chapter.

As previously mentioned, the most important capability of a VUI is to recognize voice commands, process them, and initiate the desired action^18^. We used both GTS and AutoIt to accomplish this task. Usually, GTS is able to record speech of a certain language to translate it into any other language. Also, GTS can read an inserted text aloud. For example, the voice output function can be used to learn the pronunciation of the translated text. We used this function as a speech-to-text and a text-to-speech translator to enable the interface between voice input and a processable text in both directions.

**Figure 1:**
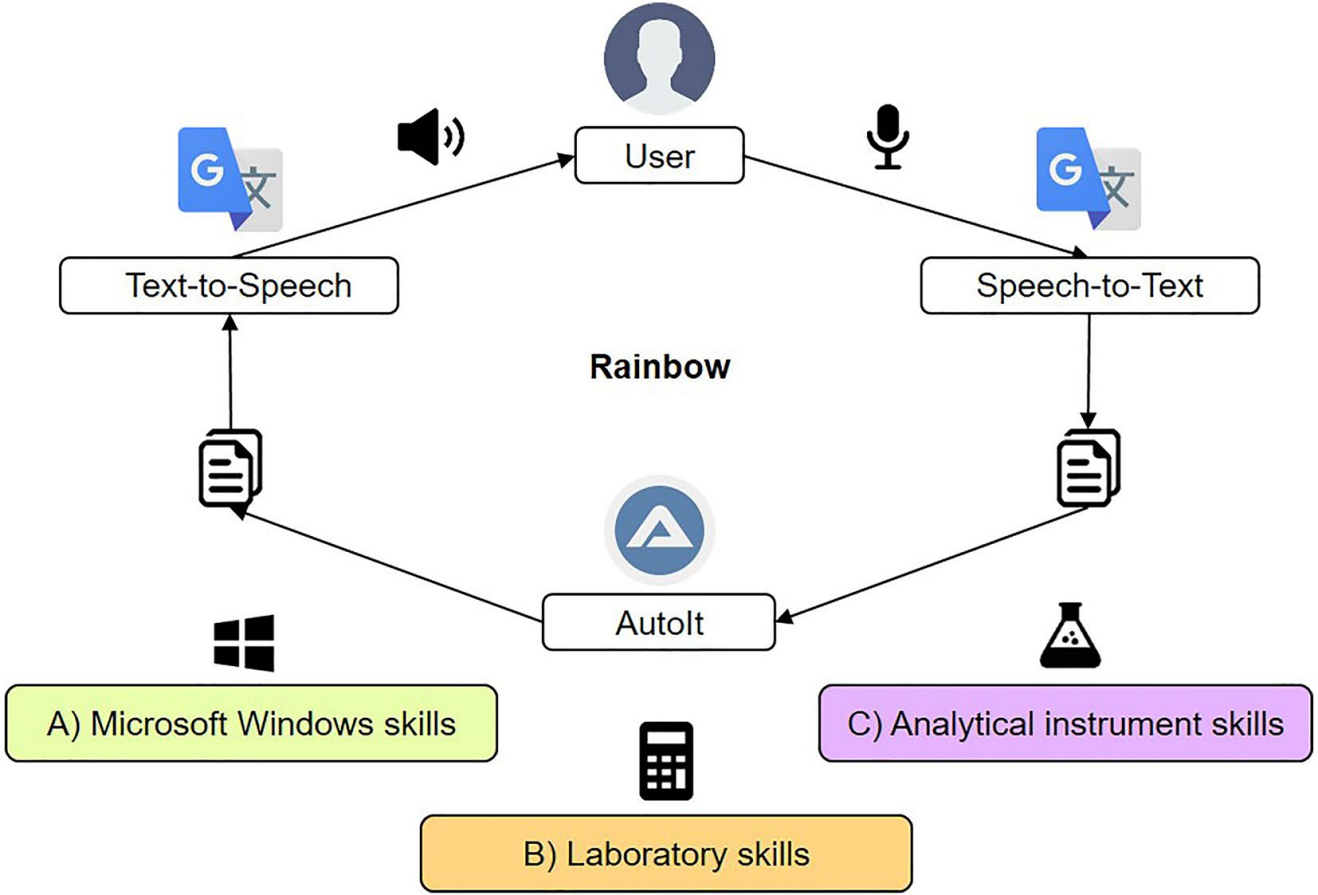
Voice command flow in voice user interface (VUI) Rainbow. The user enters a speech command which is speech-to-text translated by Google Translate Site (GTS). AutoIt puts the translated command into the clipboard for further processing. Depending on the command of the area A–C, AutoIt triggers the corresponding action, sends a Feedback into the clipboard and pastes it into GTS. GTS provides the feedback via text-to-speech translation as voice output to the user. A feedback can be further necessary questions or information about the completed task.

AutoIt handles all other tasks, including the control of GTS (e.g., start the voice recording or playback of voice output), process the voice command, and performs the corresponding action. Figure 1 shows the process flow of Rainbow. While voice recording takes place by the user, GTS translates the voice command into a text (speech-to-text, STT) and displays it in GTS. AutoIt saves the text in the clipboard for its processing. Here, AutoIt identifies and executes the relating task. A feedback as voice output either informs the user about the successful execution of the activity or asks for further information that are necessary for the chosen task.

For this purpose, AutoIt sends the text for the voice output to the clipboard, pastes it into GTS, and starts the read out for the voice output (Text-to-speech).

As mentioned above, Rainbow’s skills include functions that are supposed to support laboratory employees while working in the laboratory. The sets of skills are grouped into three areas (A-C). Area A includes Microsoft Windows automation with general activities, such as starting and closing programs, opening documents, displaying the desktop or document files, manipulating windows (e.g., minimizing or maximizing a window), handling content (e.g., copying and cutting text, saving and printing content), and researching terms on the Internet. Laboratory-specific functionalities can be found in area B "Laboratory skills". Here, the user is able to dictate notes, have step-by-step protocol read outs, or perform various scientific calculations. We also included the AutoIt timer from Wakillon (2011)^20^ to allow the user to set a timer for a certain number of minutes. Rainbow can be extended as desired, we demonstrated this in area C "Analytical Instrument Skills". We integrated a set of tasks in a separate AutoIt script that enables the user to control the spectrometer SpectraMax iD5 via its Software SoftMax Pro by speech.

### Communication with Rainbow

**Figure 2:**
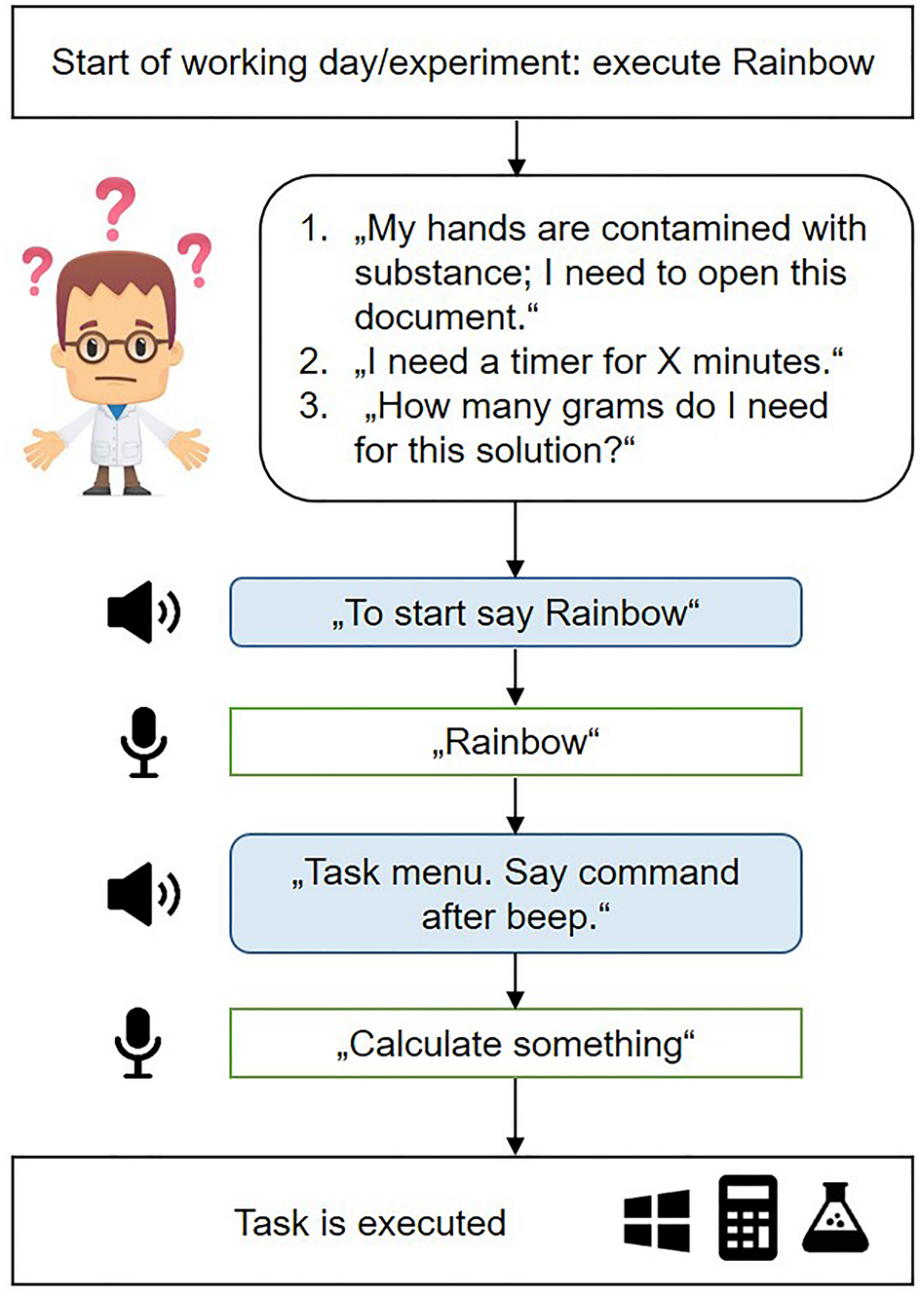
Schematic dialog between rainbow voice assistant and lab employee. Rainbow is built on the principle of question–answer logic. The user runs Rainbow manually, but can activate the voice user interface at any time by saying the triggering word "Rainbow". This activates the task menu and the user is able to enter any command among the three areas of Microsoft windows, laboratory and analytical instrument skills.

The communication between Rainbow and a user is characterized as a question–answer model, with the user's voice recorded only after a "beep” signal. Each command consists of at least two words. In most cases, the first word refers to an action (e.g., "open", "activate", "search") and the second word refers to a window or program (e.g., "Microsoft Word", "Microsoft Excel", "Timer"). Rainbow grants the user a second try if the command was not correctly recognized by GTS. The user can start Rainbow by double-clicking the executable AutoIt file "Rainbow_2.exe" manually on the Windows PC (e.g., before starting an experiment) (Fig. 2).

An initial voice output informs the user that the communication process can be started at any time by the activation word "Rainbow". As soon as Rainbow is triggered, the user enters into Rainbow’s task menu with a voice feedback. In addition to the three subject areas (area A-C), the commands are also assigned to different levels. Level 1 commands include all functions that do not require any prerequisites to be executed (Table 1).

**Table 1:** Rainbow Skills (range A–C) classified according to the level of requirements.

		
Level 1. Functions can be executed anytime with no additional conditions		
“Open a file” “Execute [Program]*” “Explore my documents” “Display my desktop” “Search [Word]*” “Hide all windows”	“Narrate protocol” “Take note” “Calculate something” “Set timer”	“Softmax Pro”

Level 2. Functions require an action of level 1		
“Print [Program]*” “Maximize [Program]*” “Minimize [Program]*” “Show [Program]*” “Save [Program]*” “Keep [Program]*” “New [Program]*” “Exit [Program]*” “Type [Program]*”		“Connect device” “Open protocol” “New plate” “Save protocol” “Read plate” “Activate lid”

Level 3. Functions require an action of level 1 with some text		
“Copy [Program]*” “Cut [Program]*”		

Rainbow commands are divided into 3 levels of requirements. Level 1 commands can be accessed at any time from Rainbow's task menu. Level 2 commands requires the involved program to be active. Level 3 commands not only require the addressed programs to be active, they also need to contain an editable text. Commands belong to either Windows based tasks, scientific calculations or scientific third party applications as symbolized by the corresponding icons on top of the table.

*GUI for respective program installed in Windows.

**Any word to be searched in Microsoft Edge.

These cover for example the execution of a program (area A), the calculation command (area B) or starting the additional analytical skills with SoftMax Pro (area C). If the user gives a level 1 command from the area A or C, the corresponding action is executed by AutoIt. The user receives a corresponding notification via voice output when the action is completed. Subsequently, the user can exit the VUI or name another command (Level 1–3). For level 1 commands of area B, further dialogs between Rainbow and the user follow to finally reach the desired action. For instance, if the user calls the "Take note" command, rainbow requests to dictate the text in a next step. Level 2 commands are tasks that need requirements. Thus, commands from area A level 2 consists of a task word (e.g., “print”, “keep”, “type”) and the corresponding program or window, that requires that the target program or window exists. This also applies for the level 2 commands of area C (controlling SoftMax Pro). The user can only connect the device or activate the lid if the software SoftMax Pro is already started. Further, there are level 3 commands for area A skills. These commands allow the user to edit some text in a specific program or window. That means level 3 commands requires not only the target program or window but also some text in the specific program (e.g., a text paragraph in Microsoft Word).

### Laboratory application

Using examples, we show how Rainbow can improve the user experience in the lab. The supplemental video (Supplement AvilaVazquez et al.) shows the use of Rainbow while performing an enzyme-linked immunosorbent assay (ELISA). Another example is the performance of a sodium dodecyl sulfate–polyacrylamide gel electrophoresis (SDS-PAGE).

We selected Rainbow's skills with the goal of supporting lab work as much as possible. One skill that can find multiple applications in the lab and helps to focus on the main experiment is the narrator function (command "Narrate protocol", area B). Before starting an experiment—as with SDS-PAGE—reagents and chemicals must be prepared, often following a protocol. Rainbow can narrate a protocol step by step by calling the "narrate protocol" function. For this purpose, AutoIt takes the protocol from a text file, with the step by step instructions separated by semicolons; and provides them stepwise to the GTS for voice output. After each instruction step, the user has time to complete the instructed task and initiate the next instruction step or cancel the narrate function by voice.

Rainbow also provides a scientific calculator which, amongst other applications, can assist during buffer preparation or during other tasks performed in the lab. Calling the command “calculate something” (area B), the user can select one of the following calculations: (1) calculate the mass needed for a solution with a definite concentration, volume and molecular weight of the substance, (2) calculate the volume of stock concentrate required to achieve a dilution with a specific concentration and volume, (3) solve a rule of three, (4) calculate the final concentration of a solution after a serial dilution. Depending on the calculation, the user can select between three units in the next step (option 1 = nmol/L, option 2 = µmol/L, option 3 = mmol/L). Triggering a calculation triggers a dialog between Rainbow and the user in which Rainbow asks for the individual quantities of the calculation. Calculation option 1 can be used, for example, for the unknown amount of NaCl in a Tris-buffered saline (TBS). After selecting "option 3" for "mmol/L", Rainbow asks for the desired concentration (e.g., "0.0015"), the desired volume (e.g., "900"), and the formula weight of the substance in grams per mol ("58.44"). The result (“78.89 g”) is provided by voice output and Rainbow creates a text file with all the data of the calculation. This text file can then be processed for documentation purposes with the Area A commands (e.g., "copy editor" to "microsoft word" or "save editor"). Using the "take note" command, for example, the sequence of the sample can be documented while the gel is charged by the user. After that, the time of electrophoresis (or later the staining process) can be limited by Rainbow's timer ("set timer", area B).

### Expandable interface

Rainbow is expandable in two ways. First, the accuracy of speech recognition can be improved and second, new skills can be added.

During Rainbow’s development, we identified words of commands that GTS repeatedly misrecognized. We integrated the incorrect words into the script and linked them to the corresponding command (e.g., “safe" for "save [program]", "maximise" and "minimise" for "maximize [program] and "minimize [program]"). All additional first word commands are shown in Table S2. Depending on the English pronunciation, GTS may not correctly recognize the commands of another individual user (or users) with other dialects/pronunciation. Thus, we provide an AutoIt-based GUI to give users the option to enter misrecognized commands (e.g., “Snow”) and link them to the desired Rainbow skill (e.g., “Show [Program]”). After the user confirms the entry, AutoIt saves the association between the wrong word and the correct command in a separate Microsoft Excel file. When GTS recognizes "Snow" in the future, AutoIt will first compare the command with the integrated commands in the main script. Since this does not result in a match, AutoIt extends the search from "Snow" to the external Excel file. Here AutoIt finds the correlation between "Snow" and "Show" and finally executes the command "Show [Program]".

Due to Rainbow’s AutoIt-based structure, it can be easily extended with further functions. Thus, it is possible to connect further AutoIt scripts to the Rainbow main script. We show this here as an example with the timer function (area B) and the set of skill for the Softmax Pro software (area C). We adapted the timer script from Wakillon (2011)^20^ and stored it as a separate executable file that is started by the main script as soon as the command "set timer" is called. By the voice command "SoftMax Pro", the main script starts the corresponding linked executable file, which offers another set of skills (Table 1, area C, level 2 commands). As shown in the supplemental video (Supplementary Video S1), the user is able to control the spectrometer as an analytical instrument that has usually no option for speech recognition. Through the integration of the corresponding analysis software, the user is able to connect the device, open or save a protocol, create a new plate, open or close the lid and start the measurement by voice. With this example, we showed that it is possible to integrate single functions on the one hand (e.g., timer function), but also entire sets of skills and thus further software-based laboratory devices into Rainbow to control them by voice.

### Accuracy and reliability

As mentioned before, during the development of Rainbow, we integrated frequently misrecognized words into our AutoIt script to reduce the number of errors in Rainbow.

We wanted to test whether the accuracy of Rainbow could be significantly improved compared to the conventional GTS.

For the accuracy test, we wanted to revise how often the given commands result in success—meaning, the correct action is performed. In total, the 38 volunteers—with a range of dialects—tested 1349 Rainbow speech commands, with Rainbow acting in 1232 (91.3%) cases correct. Due to the extensibility of Rainbow in terms of misrecognized words, we wanted to compare the accuracy of Rainbow's speech recognition to the accuracy of GTS alone and test whether Rainbow's accuracy was significantly better. For the same amount (1349) of called commands only using GTS, GTS identified 1148 (85.1%) words correct. Meaning that with GTS, every 6.7 command has to be repeated, while with Rainbow it is one in 11.5. Comparing the speech recognition of Rainbow and GTS, the hypothesis test showed that the recognition accuracy of rainbow is significantly higher than the accuracy of GTS (p = 7.292e−09). This means that Rainbow's strategy of relating misrecognized words to correct functions has not only led to subjective improvement but also statistically proven. Please see supplementary Table S3 for raw data.

**Figure 3:**
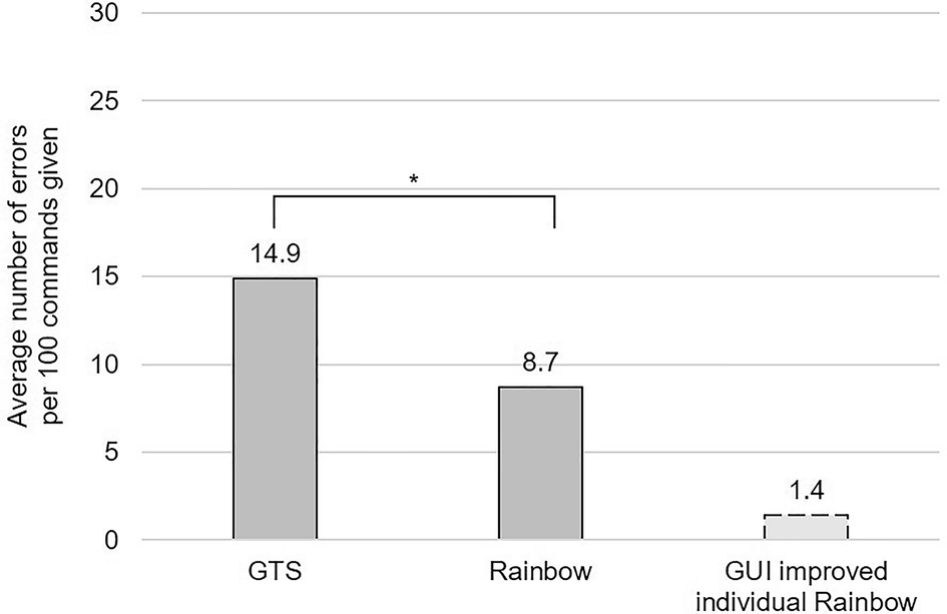
Average number of errors per 100 commands given. The results of the accuracy tests showed that with GTS alone 14.9 of 100 commands were misrecognized; while with Rainbow on average 8.7 of 100 command were misrecognized. The McNemar hypothesis test confirms the significant p value of these results (*p = 7.292e−09). As an example, we were able to show with an individual employee that the use of the GUI further improves the accuracy. This individual can expect an average of 1.4 errors for 100 commands.

Next, we wanted to find out the benefit of the GUI in terms of accuracy. To do this, we conducted further accuracy tests with one employee as an example. Rainbow showed 95.8% accuracy for the employee with three of 71 spoken commands misrecognized. We then used GTS to identify commands that Rainbow did not correctly recognize for this employee with. From these, 9 misrecognized commands were relevant to Rainbow's function. The employee implemented these 9 terms and 3 misrecognized commands coming from the initial rainbow accuracy test in Rainbow via the GUI, which required 4 min of time. The subsequent accuracy test showed that the employee had an accuracy of 98.6%. This means that the user had to repeat every 23.8 commands when using the original Rainbow and every 71.4 commands when using Rainbow after the GUI improvement. Figure 3 demonstrates the average of errors per 100 commands for GTS, Rainbow and the GUI improved individual Rainbow VUI. Raw data of this test can be viewed at supplementary Table S4.

## Discussion

With the help of the free software components AutoIt and GTS, we were able to establish a voice assistant for life science laboratories. Rainbow's architecture enables flexibility, data protection, and improved accuracy. The user can activate Rainbow by a triggering word—a main concern for user privacy as known in commercial voice assistants like Apple’s Siri or Amazon’s Alexa^21^. We also see further advantages of Rainbow in terms of data privacy. As with voice assistants based on commercial services (such as Amazon's Alexa), sensitive data are stored on external servers and could be a target for possible hacking attacks^2^. GTS is also known to store the translated texts on Google's servers^22^. However, this presents a risk only for the dictation function, since the information enters in a GTS here. All other voice commands also pass GTS, but usually do not contain any critical data. Further processing of the commands takes place via AutoIt on the local desktop PC. However, Rainbow is not operational in laboratories that do not have Internet access on the PC due to increased data security. Here it would be conceivable to use the offline version of the GTS via an Android emulator. However, this can lead to a reduced accuracy using the offline version^14^.

Rainbow owns various sets of commands. We chose the integrated skills to be as broadly applicable as possible in the bioscience laboratory area and to improve communication between the laboratory employees and hardware and software components in the laboratory. Next to general Windows functions, specific scientific skills such as the scientific calculator or the timer can be helpful when gloves are dirty or work at the lab bench should not be interrupted. Unfortunately, Rainbow is quite inflexible regarding the scientific calculator. For instance, it is possible so calculate the weighing mass for a certain solution with given molecular weight, desired volume, and concentration, but it is not possible to change this formula to calculate the final volume. Of course, several variations of formulas could be integrated into Rainbow, while a simpler solution—such as integrating an external scientific calculator—would also be conceivable.

A particular strength of Rainbow is its adaptability. By integrating the existing AutoIt Timer, we show that Rainbow can be extended with additional features.

We also demonstrated, that third-party software, such as analysis software, can be integrated into Rainbow, enabling voice control of analysis devices (SoftMax Pro). While these extensions require programming expertise from AutoIt, our experience shows that AutoIt is a very easy programming language to learn. Thus, with approximately 10 h of AutoIt experience, our life science students can understand and work with Rainbow's main script.

Our study showed that the detection accuracy of Rainbow is significantly (p = 7.292e−09) higher than that of GTS alone. The underlying McNemar test was suitable for this examination since our data are two related samples (same users speaking the same commands in GTS and Rainbow) and only distinguish between correct (1) and incorrect (2) (dichotomous characteristics).

Further, our accuracy tests showed that with GTS every 6.7 command and with Rainbow every 11.5 command is misrecognized, which means the repetition rate of GTS is only about half as good as that of Rainbow. Since we used several volunteers with a wide variety of dialects for the accuracy test, we cannot compare the results with other VUI like from Austerjost et al.^2^.

Besides Rainbow's accuracy, individual users have the opportunity to improve the precision of their personalized Rainbow VUI. Using the GUI, wrongly recognized words can be easily integrated into Rainbow. We were able to show that with minimal effort, one employee could improve the own Rainbow's repetition rate from 23.8 to 71.4, which is the threefold improvement. Again, we would like to mention that this score is an individual—and therefore exemplary—result, which means that other users may achieve varying outcomes.

We could not identify any consistent patterns in the misrecognized words. GTS exhibited difficulty recognizing both single-syllable commands (e.g., "show," "new") and multi-syllable commands (e.g., "Minimize," "Narrate"). Errors were diverse, ranging from those occurring frequently among multiple subjects (e.g., "Minimise" instead of "Minimize," "Safe" instead of "Save") to individual errors, as illustrated in our GUI test (e.g., "auction" instead of "option").

With the GUI, it is possible not only to improve accuracy, but also to create shortcuts (e.g. "1" corresponds to "calculate something"). Austerjost et al.^2^ demonstrated a VUI that is intuitive to control, meaning that several terms or synonyms results in the same action. With the help of the GUI, users can improve Rainbow regarding the intuitive control. We have exemplified this with the commands "Exit" and "Escape", which both results in closing the target window (see Table S2). This allows each user or laboratory to add their own commands and shortcuts to Rainbow without any programming knowledge. Nevertheless, attention should be paid to the additional words that are entered into the GUI. For example including the term "you" (played) for the command "new" (plate) means that an user could never add a new command named "you". Therefore, new words for the GUI should always be excluded from other laboratory activities. However, during the development and test phase, there have never been any restrictions due to this theoretical limitation.

During the testing phase, we realized that the use of Rainbow requires some practice. Difficulties were faced when users were not familiar with the command structure, commands were not known, or the timing of voice input was unsuitable. This can lead to employee frustration and even aversion to voice assistants^6^. Thus, it is helpful to provide a “cheat sheet” with all commands in the laboratory. Users should train the speech commands as given in the cheat sheet. This may seem like a limitation, however the speech commands designed for Rainbow are intuitive because of the “task + program” structure. All 38 test users became familiar with Rainbow´s speech command structure within minutes to approximately an hour. In addition, a brief introduction about Rainbow would surely improve the user’s performance with the VUI.

As the participants solely read Rainbow's voice commands during the tests, they were unable to provide any further evaluation of Rainbow. A comprehensive assessment of Rainbow's suitability can be conducted once it is established for testing in laboratory routine.

We want to point out that GTS, if set to English, can only reliably recognize English terms. For example, it is not possible to start software or open files with Rainbow that have a name in another language. Rainbow is open source available at https://github.com/LabAutoSig/Rainbow_V2.0 and is not associated with any purchasable components. However, we recommend the use of a Bluetooth microphone for communication with Rainbow. This will ensure Rainbow's accuracy and allow the user to move freely in the laboratory. Additionally, the user does not interfere with other staff talking to Rainbow, and is not disturbed by colleagues or noise in the laboratory.

### Supplementary Information

Supplementary Table S1.

Supplementary Table S2.

Supplementary Table S3.

Supplementary Table S4.

Supplementary Video S1.

## Data Availability

All data generated or analyzed during this study are included in this published article and its Supplementary Information files. The raw data can be viewed in Supplementary Table S3 and Supplementary Table S4.
