# Supplementary material for: An expandable voice user interface as lab assistant based on an improved version of Google’s speech recognition
Source: Sci Rep. 2023 Nov 9;13:19451. doi: 10.1038/s41598-023-46185-x (PMC10636177; doi:10.1038/s41598-023-46185-x)
Supplement: Supplementary file 1 — Supplementary Table S1. [file 41598_2023_46185_MOESM1_ESM.docx]

**Table S1. Set of commands for accuracy testing of rainbow and GTS.** The sets consist of 35 (set 1) or 36 (set 2) Rainbow commands that lead to a meaningful conversation with Rainbow. When testing Rainbow, the volunteer speaks command 1 to 35 or 36 at the appropriate time and receives a voice output from Rainbow for each voice input (listet to the right of the corresponding command). For the accuracy of GTS, the same commands were recorded, however, there is no response from the system.
* Example voice input for any text that Rainbow requires at this point. Since this voice input is not a specific command, it is not considered for the accuracy test and is not used for the GTS test.

| **Set 1** | | | **Set 2** | |
| --- | --- | --- | --- | --- |
| **No.** | **Command** | **Voice output Rainbow** | **Command** | ***Voice output Rainbow*** |
|  |  | *To start say Rainbow.* |  | *To start say Rainbow.* |
| 1. | Rainbow | *Rainbow Task menu. Say command after beep.* | Rainbow | *Rainbow Task menu. Say command after beep.* |
|  |  |  |  |  |
| 2. | Explore My Documents | *Displaying Rainbow documents. Say next command. Otherwise, say cancel.* | Display My Desktop | *Desktop ready. Say next command. Otherwise, say cancel.* |
|  |  |  |  |  |
| 3. | Narrate protocol | *Listen to protocol first step?* | Open a file | *Which document?* |
|  |  |  |  |  |
| 4. | Yes | *Next step? Say yes, no, or exit.* | List of materials | *Document open. Say next command. Otherwise, say cancel.* |
|  |  |  |  |  |
| 5. | Yes | *Next step? Say yes, no, or exit.* | Type Microsoft Word | *What would you like to type in Word?* |
|  |  |  |  |  |
|  |  |  |  |  |
|  |  |  | * This is some text for rainbow | *New text in Word. Say next command. Otherwise, say cancel.* |
| 6. | Exit | *Closing Narrator. Say next command. Otherwise, say cancel.* | Cut Microsoft Word | *Cut function done in Word. Say next command. Otherwise, say cancel.* |
|  |  |  |  |  |
| 7. | Take note | *[beep sound]* | Exit Microsoft Word | *Before exiting Word. Save [Document – Word]? Say yes, no or cancel to stop from exiting.* |
|  |  |  |  |  |
|  | * This is some text coma Rainbow can read protocols out loud coma calculate a rule of three coma set a timer coma or open a file dot SoftMax Pro can also be included in Rainbow’s tasks dot | *Note copied in Word. Saved in Rainbow folder. Say next command. Otherwise, say cancel.* |  |  |
|  |  |  |  |  |
| 8. | New Microsoft Word | *New document in Word. Say next command. Otherwise, say cancel.* | No | *File not saved. Program closed. Say next command. Otherwise, say cancel.* |
|  |  |  |  |  |
| 9. | Calculate something | *Listen to all calculator options? Say yes, or, say "option" and the option number.* | Calculate something | *Listen to all calculator options? Say yes, or, say "option" and the option number.* |
|  |  |  |  |  |
| 10. | Option 1 | *Select calculation units. Option 1: nano mol per liter. Option 2: micro mol per liter. Option 3: milli mol per liter. Please say "option" and option number to continue.* | Option 3 | *Based on this sentence: If Alpha corresponds to Beta and Gamma corresponds to Detla. Which is your unknown value?* |
|  |  |  |  |  |
| 11. | Option 1 | *Provide desired concentration in nanomole per liter.* | Gamma | *Provide your Alpha value* |
|  |  |  |  |  |
| 12. | 300 | *Provide desired volume in ml.* | 24 | *Provide your Beta value* |
|  |  |  |  |  |
| 13. | 250 | *Provide formula weight of your solution in grams per mol.* | 8 | *Provide your Delta value* |
|  |  |  |  |  |
| 14. | 58.44 | *You need [0.00000438] grams. Say next command. Otherwise, say cancel.* | 5 | *Missing value [15]. Say next command. Otherwise, say cancel.* |
|  |  |  |  |  |
| 15. | Calculate something | *Listen to all calculator options? Say yes, or, say "option" and the option number.* | Calculate something | *Listen to all calculator options? Say yes, or, say "option" and the option number.* |
|  |  |  |  |  |
| 16. | Option 2 | *Select calculation units. Option 1: nano mol per liter. Option 2: micro mol per liter. Option 3: milli mol per liter. Please say "option" and option number to continue.* | Option 4 | *Select calculation units. Option 1: nano mol per liter. Option 2: micro mol per liter. Option 3: milli mol per liter. Please say "option" and option number to continue.* |
|  |  |  |  |  |
| 17. | Option 2 | *Provide stock concentration in micromole per liter.* | Option 3 | *Provide stock solution concentration in millimole per liter.* |
|  |  |  |  |  |
| 18. | 150 | *Provide desired final volume in milliliters.* | 300 | *Provide the number of times you will dilute* |
|  |  |  |  |  |
| 19. | 50 | *Provide desired final concentration in [micromole per liter].* | 10 | *Provide the volume dilution taken from stock solution in mililiters.* |
|  |  |  |  |  |
| 20. | 75 | *You need [25] ml. Say next command. Otherwise, say cancel.* | 0.1 | *Provide the desired final volume of each dilution in mililiters.* |
|  |  |  |  |  |
| 21. | Copy Editor | *Where would you like to paste the copied contents?* | 0.5 | *Final concentration [0.00003072 millimole per liter]. Say next command. Otherwise, say cancel.* |
|  |  |  |  |  |
| 22. | Microsoft Word | *Text copied in Word. Say new command. Otherwise, say cancel.* | Keep Editor. | *Say new file name.* |
|  |  |  |  |  |
| 23. | Minimize Microsoft Word | *Word minimized. Say next command. Otherwise, say cancel.* | Serial dilution calculation | *File saved. Say next command. Otherwise, say cancel.* |
|  |  |  |  |  |
| 24. | Set timer | *Please set your timer: say number of minutes and the word minutes.* | Save Editor | *File saved. Say next command. Otherwise say cancel.* |
|  |  |  |  |  |
| 25. | 1 minute | *You set a timer to 1 minute [Pause for a minute] Timer ended. Say next command. Otherwise, say cancel.* | Execute Microsoft Excel | *Excel open. Say next command. Otherwise say cancel.* |
|  |  |  |  |  |
| 26. | SoftMax Pro | *Please say command for SoftMax Pro.* | Maximize Editor | *Editor maximized. Say next command. Otherwise say cancel.* |
|  |  |  |  |  |
| 27. | Open protocol | *Say protocol name to open.* | Print Editor | *You chose the print task. To confirm say yes. Otherwise, say cancel.* |
|  |  |  |  |  |
| 28. | Test for Rainbow | *Protocol open. Say next command. Otherwise, say cancel.* | Cancel | *Print function canceled. Say next command. Otherwise say cancel.* |
|  |  |  |  |  |
| 29. | Connect Device | *Device connected. Say next command. Otherwise, say cancel.* | Show Editor | *Editor window activated. Say next command. Otherwise say cancel.* |
|  |  |  |  |  |
| 30. | New Plate | *New plate. Say next command. Otherwise, say cancel.* | SoftMax Pro | *Please say command for SoftMax Pro.* |
|  |  |  |  |  |
| 31. | Activate Lid | *Active drawer. Say next command. Otherwise, say cancel.* | New plate | *New plate. Say next command. Otherwise say cancel.* |
|  |  |  |  |  |
| 32. | Cancel | *Rainbow task menu. Say next command. Otherwise, say cancel.* | Read plate | *Plate ready. Say next command. Otherwise say cancel.* |
|  |  |  |  |  |
| 33. | Search Immunoassay | *Searching for Immunoassay. Review search in Microsoft Edge. Say next command. Otherwise, say cancel.* | Save protocol | *Say new file name* |
|  |  |  |  |  |
| 34. | Hide all windows | *All your windows are minimized. Say next command. Otherwise, say cancel.* | New experiment | *Protocol saved. Say next command. Otherwise say cancel.* |
|  |  |  |  |  |
| 35. | Cancel | *Goodbye.* | Cancel | *Rainbow task menu. Say next command. Otherwise say cancel.* |
|  |  |  |  |  |
| 36. | - | *-* | Cancel | *Goodbye.* |
|  |  |  |  |  |
