# Supplementary material for: An expandable voice user interface as lab assistant based on an improved version of Google’s speech recognition
Source: Sci Rep. 2023 Nov 9;13:19451. doi: 10.1038/s41598-023-46185-x (PMC10636177; doi:10.1038/s41598-023-46185-x)
Supplement: Supplementary file 2 — Supplementary Table S2. [file 41598_2023_46185_MOESM2_ESM.docx]

**Table S2. Improved accuracy based on Rainbows autocorrect function.** In addition to the correct commands, the base version of Rainbow includes a set of potentially incorrectly recognized commands. These ─ by Google Translate (GTS) ─ misrecognized terms were identified during development and implemented into the Rainbow script. Therefore, these incorrect commands lead to the correct event when Rainbow is applied, even if they were misrecognized by GTS. This condition is the basis for the improved recognition rate of Rainbow compared to GTS.

| **Rainbow command** | **Recognized by Rainbow as correct command** |
| --- | --- |
| Open [a file] | Oven |
| Execute [Program] | Executed |
| Explore [my documents] | Explorer |
| Display [my Desktop] | Displaying |
| Hide [all windows] | Hyatt, Height, Hi |
| Maximize [Program] | Maximise, Maximus, Maximiles |
| Minimize [Program] | Minimise, Minnie (Mouse) |
| Show [Program] | Sean |
| Save [Program] | Safe, Face, Shape |
| Keep [Program] | Skip, Cheap |
| New [program] | You, Knew, Nail |
| Exit [Window] | Exits, Except, **Escape, Escaped** |
| Type [Program] | Time |
| Copy [Program] | Copying, Call, Coffee |
| Cut [Program] | Hot, Contact, Go, Case, Cat, Caught, Cup |
| Narrate [protocol] | Not, Marriage, Married, Merit, Never, Now, Navigate, Madrid, Mairead, My |
| One | 1 |
| Two | 2 |
| Three | 3, free |
| Four | 4, for |
| Beta | PETA, Pizza, betta, Peter, better |
| Set [timer] | Sent |
| SoftMax | Soft, Stuffed |
| Open [Protocol] | Oven |
| New [plate] | You, Knew, Nail |
| Save [Protocol] | Safe, Face, Shape |
| Read [plate] | Reach |
| Activate [lid] | Actuate |
